# Effects of explant size on epithelial outgrowth, thickness, stratification, ultrastructure and phenotype of cultured limbal epithelial cells

**DOI:** 10.1371/journal.pone.0212524

**Published:** 2019-03-12

**Authors:** O. A. Utheim, L. Pasovic, S. Raeder, J. R. Eidet, I. G. Fostad, A. Sehic, B. Roald, M. F. de la Paz, T. Lyberg, D. A. Dartt, T. P. Utheim

**Affiliations:** 1 Department of Medical Biochemistry, Oslo University Hospital, Oslo, Norway; 2 Institute of Clinical Medicine, Faculty of Medicine, University of Oslo, Oslo, Norway; 3 Norwegian Dry Eye Clinic, Oslo, Norway; 4 Department of Ophthalmology, Oslo University Hospital, Oslo, Norway; 5 Department of Oral Biology, Faculty of Dentistry, University of Oslo, Oslo, Norway; 6 Department of Maxillofacial surgery, Oslo University Hospital, Oslo, Norway; 7 Department of Pathology, Oslo University Hospital, Oslo, Norway; 8 Institut Universitari Barraquer, Universitat Autonoma de Barcelona, Barcelona, Spain; 9 Schepens Eye Research Institute/Massachusetts Eye and Ear Infirmary, Department of Ophthalmology, Harvard Medical School, Boston, MA, United States of America; 10 Department of Plastic and Reconstructive Surgery, Oslo University Hospital, Oslo, Norway; 11 Department of Ophthalmology, Stavanger University Hospital, Stavanger, Norway; 12 Department of Clinical Medicine, Faculty of Medicine, University of Bergen, Bergen, Norway; 13 Department of Ophthalmology, Soerlandet Hospital Arendal, Arendal, Norway; 14 Department of Ophthalmology, Drammen Hospital, Vestre Viken Hospital Trust, Drammen, Norway; 15 National Centre for Optics, Vision and Eye Care, Faculty of Health and Social Sciences, University of Southeast Norway, Kongsberg, Norway; Oklahoma State University Center for Health Sciences, UNITED STATES

## Abstract

**Purpose:**

Transplantation of limbal stem cells is a promising therapy for limbal stem cell deficiency. Limbal cells can be harvested from either a healthy part of the patient’s eye or the eye of a donor. Small explants are less likely to inflict injury to the donor site. We investigated the effects of limbal explant size on multiple characteristics known to be important for transplant function.

**Methods:**

Human limbal epithelial cells were expanded from large versus small explants (3 versus 1 mm of the corneal circumference) for 3 weeks and characterized by light microscopy, immunohistochemistry, and transmission electron microscopy. Epithelial thickness, stratification, outgrowth, ultrastructure and phenotype were assessed.

**Results:**

Epithelial thickness and stratification were similar between the groups. Outgrowth size correlated positively with explant size (*r* = 0.37; P = 0.01), whereas fold growth correlated negatively with explant size (r = –0.55; P < 0.0001). Percentage of cells expressing the limbal epithelial cell marker K19 was higher in cells derived from large explants (99.1±1.2%) compared to cells derived from small explants (93.2±13.6%, *P* = 0.024). The percentage of cells expressing ABCG2, integrin β1, p63, and p63α that are markers suggestive of an immature phenotype; Keratin 3, Connexin 43, and E-Cadherin that are markers of differentiation; and Ki67 and PCNA that indicate cell proliferation were equal in both groups. Desmosome and hemidesmosome densities were equal between the groups.

**Conclusion:**

For donor- and culture conditions used in the present study, large explants are preferable to small in terms of outgrowth area. As regards limbal epithelial cell thickness, stratification, mechanical strength, and the attainment of a predominantly immature phenotype, both large and small explants are sufficient.

## Introduction

Limbal stem cell deficiency is a potentially blinding condition characterized by painful epithelial defects in the cornea due to insufficient function or total loss of the corneal epithelial stem cell population. These stem cells are located in the transitional zone between the transparent cornea and the conjunctiva, called the limbal region. Limbal stem cells give upon activation rise to rapidly proliferating daughter cells, called transit-amplifying cells which in turn can mature into terminally differentiated cells localized in the suprabasal layers of the corneal epithelium [1]. Limbal stem cell deficiency can be caused by a multitude of factors, including genetic, e.g. aniridia, or acquired, e.g. infections, chemical burns, and autoimmune diseases [2].

A number of surgical treatments have been explored in order to restore the limbal stem cell population. One well documented and established method is transplantation of *ex vivo* expanded autologous limbal epithelial cells (LEC), first coined by Pellegrini and coworkers [3], where a limbal explant from a healthy region of the patient’s eye is harvested and expanded in the laboratory by culturing it on a substrate, e.g. fibrin [4] or human amniotic membrane (HAM) [5]. When the explant is sufficiently expanded to cover the entire cornea and the limbal region, the limbal epithelial cell sheet is transplanted to the diseased eye. In 2012, Sangwan and coworkers introduced simple limbal epithelial transplantation (SLET), a novel method in which a 4 mm^2^ strip of donor limbal tissue is divided into small pieces before being distributed over an amniotic membrane placed on the cornea [6]. This technique does not include *ex vivo* cultivation, thereby reducing costs and preparation time by circumventing the need for EU approved, specialized cell culture laboratories. The size of the limbal explant obtained is the smallest yet reported for limbal autograft transplantation without *ex vivo* cultivation.

Other approaches to treat total limbal stem cell deficiency without *ex vivo* expansion are direct grafting of limbal grafts, either autografts from the healthy contralateral eye (conjunctival limbal autografts–CLAU) [7] or allografts from cadaveric or [8, 9] or living-related donors [10] (keratolimbal allografts (KLAL) or living-related conjunctival allografts (LR-CLAL). The size of the living-related donor explant to treat limbal stem cell deficiency has been gradually reduced over the years. In Kenyon and Tseng’s pioneering work, three clock-hours, equaling approximately 9 mm of the limbus, was excised from the donor eye [11]. Later protocols employed explants measuring two to four clock-hours (6–12 mm) [12], two to three clock-hours (6–9 mm) [13, 14], and finally the minimal conjunctival limbal autograft proposed by Kheirkhah *et al*. [15], measuring two clock-hours (6 mm). Nevertheless, KLAL and LR-CLAL have limited long-term success and complications have been observed, such as infections and rejection [12, 16]. Large biopsies are associated with a higher donor complication rate [17]. In the worst case, infection and destruction of the donor corneal epithelium can occurs [12, 18].

Smaller cadaveric limbal explants can also be cultured to produce LEC sheets to cover the cornea in cases where autologous or living-related donor tissue is not available. This approach might reduce the high complication rate with KLAL. There have not, however, been any published clinical studies using cultured cadaveric LEC.

There are two main methods for producing *ex vivo* cultured LEC for transplantation: the dissociated cell culture system and the explant culture system [19]. In the dissociated cell culture system, single LEC are released from the limbal explant after enzymatic treatment [20]. The LEC are thereafter seeded onto the carrier in the presence of a feeder layer of growth arrested murine fibroblasts (3T3 feeder cells). In the explant culture system, the limbal biopsy is pre-treated with dispase for a few minutes, enough for the explant to attach to the surface of the carrier, and then submerged in the culture medium. Both cultivation strategies produce epithelial sheets with a corneal phenotype suitable for transplantation [21]. An advantage of the explant culture is that it can be cultured without a 3T3 feeder layer, and hence avoid contamination with xenobiotics. However, the cell suspension technique has been shown to be superior to the explant culture technique in terms of producing an undifferentiated epithelium [22] with more desmosome junctions that protect against postoperative complications [21]. Moreover, Kolli *et al*. revealed a decline in stem cell properties in the outgrowth with increasing distance from the explant [23].

Hence, the explant method needs to be further studied and optimized. An initial step is to determine the optimal size of the explant, which is important not only in the explant culture technique, but also in the non-culture SLET technique. The explant size has implications both for the donor site and for the recipient eye. As regards to the donor site, a small explant is clearly beneficial in order to reduce the risk of complications [24]. For the recipient, a small explant may be beneficial in order to ensure a smooth ocular surface after transplantation. In addition, small samples will utilize the donor material more efficiently, possibly yielding more than one culture per explant obtained from the donor. It is clearly beneficial to start more than one culture prior to LEC transplantation, since growth failure happens in 20% of cases with live donors [25] and 40–50% of cases with cadaveric donors [25–27]. Nevertheless, the explant needs to be large enough to contain the necessary stem cells and proliferative potential to restore the limbal region and the corneal epithelium.

In 2017, Kethiri *et al*. compared biopsies at different sizes between 0.3 and 2 mm^2^, from both cadaveric and live donors, and found that biopsies down to 0.3 mm^2^ for live tissue and ≥ 0.5 mm^2^ for cadaveric tissue gave sufficient outgrowth to cover the cornea after 8 days of culture. However, in Kethiri *et al*.*’s* study the maximum biopsy was 2 mm^2^ while the size of the limbal explants used for *ex vivo* expansion varies in the literature from 1 mm [3] to 3 mm [28] of the corneal circumference. Hence, we wanted to investigate a cadaveric model of human limbal epithelial cell sheet (LEC) cultured on HAM with explants of 1 mm versus 3 mm of the corneal circumference. In the current study, we sought to investigate the effect of explant size on several traits known to be important for transplant function, such as epithelial thickness, stratification, outgrowth, ultrastructure, and phenotype.

In 2012, Eidet *et al*. studied conjunctival biopsies and found that small conjunctival biopsies grew more per explant area–that is, with a higher fold growth–than large biopsies, even if the large biopsies achieved a higher total outgrowth area [29]. Hence, in the present study, we also calculated fold growth as outgrowth area/explant area to see whether the same observation applied to LEC.

## Materials and methods

### Supplies

Phosphate-buffered saline (PBS), Dulbecco’s Modified Eagle’s Medium (DMEM), 4-(2-hydroxyethyl)-1-piperazineethanesulfonic acid (HEPES) buffer, Ham’s F12 solution, fetal bovine serum (FBS), sodium bicarbonate, dimethyl sulphoxide (DMSO), human epidermal growth factor, hydrocortisone, insulin-transferrin-sodium selenite medium supplement, gentamicin, and amphotericin B were purchased from Sigma-Aldrich (St. Louis, MO). Dispase II was obtained from Roche Diagnostics (Basel, Switzerland), cholera toxin A subunit from Biomol (Exeter, UK), 5 mm biopsy punches from Kai Industries (Gifu, Japan), 6–0 C-2 monofilament sutures (Ethicon Ethilon) from Johnson & Johnson (New Brunswick, NJ), 24 mm culture plate inserts (74 μm mesh size polyester membrane; Netwell) from Corning Costar (Corning, NY), and vancomycin from Abbott Laboratories (Abbott Park, IL). The antibodies used in the study are described in Table 1.

**Table 1:** Antibodies used in the present study.

Antigen	Dilution	Clone	Company
**K19**	1:20	Mouse, monoclonal RCK108	DAKO, Agilent Technologies, Santa Clara, CA, USA
**ABCG2**	1:20	ABCG2 protein. Mouse, monoclonal clone bxp-21.	Sigma Aldrich, St. Louis, MO, USA
**β1 Integrin**	1:10	Mouse anti-integrin-1 antibody (clone 7F10)	Novocastra Laboratories Ltd., Leica Biosystems, Newcastle upon Tyne, UK
**p63**	1:25	p63 protein mouse monoclonal clone bxp-21.	DAKO Cytomation Norden A/S, Glostrup, Denmark
**p63α**	1:200		Primm, Milan, Italy
**K3**	1:500	K3, Clone AE5 Mouse anti-cytokeratin	ImmuQuest, Cleveland, UK
**Connexin 43**	1:500	Rabbit, polyclonal, C6219	Sigma Aldrich, St. Louis, MO, USA
**E-cadherin**	1:25	Mouse anti-E cadherin	Novocastra Laboratories Ltd., Leica Biosystems, Newcastle upon Tyne, UK
**Ki67**	1:75	Ki67 Mouse monoclonal MIB-1	DAKO, Agilent Technologies, Santa Clara, CA, USA
**PCNA**	1:3500	PCNA. Mouse monoclonal, M879	DAKO, Agilent Technologies, Santa Clara, CA, USA

### Preparation of cadaveric limbal explants and culture of LEC sheets

The research was conducted in accordance with the Declaration of Helsinki and approved by the Norwegian Regional Committee for Medical and Health Research Ethics. Corneoscleral tissue from three donors (five eyes) was obtained from El centro de Oftalmología Barraquer (Barcelona, Spain) after informed, written consent from the next of kin for the usage of tissue for research purposes. The corneoscleral rings were harvested shortly after death, and the superior part was marked by a surgical suture in the extreme scleral edge. Right versus left eyes were placed in marked containers with organ culture medium at ambient temperature and shipped to the laboratory at Oslo University Hospital, Oslo, Norway where the experiments were performed. Hence, the limbal rings could be oriented with respect to superior/inferior/nasal/temporal part upon arrival in the laboratory. In order to avoid variations in stem cell density between the groups based on limbal harvesting site, only the superior and inferior parts of the limbal rings were used.

At Oslo University Hospital, HAM were donated after informed, written consent from women after elective caesarian section. The HAMs were prepared and cryopreserved as previously reported [30], thereafter thawed and attached to the polyester membrane of Netwell culture plate inserts (Corning, New York, USA) using 6–0 non-absorbable sutures. The limbal explants were prepared and cultured onto amniotic membranes as previously described by Meller *et al* [31]. Briefly, the tissue was rinsed three times with DMEM containing 50 μg/mL gentamicin and 1.25 μg/mL amphotericin B. After careful removal of excessive sclera, conjunctiva, iris, and corneal endothelium, the remaining tissue was placed in a culture dish and exposed for 10 minutes to Dispase II in Mg- and Ca-free Hanks’ balanced salt solution, at 37°C under humidified 5% carbon dioxide, and thereafter rinsed with DMEM containing 10% FBS in order to inhibit the Dispase activity. Next, the entire circumference of the limbal rings were divided into 24 pieces of approximately 1 mm x 8 mm (small explants) and 24 pieces of approximately 3 mm x 8 mm (large explants) by the use of a surgical steel blade and a ruler, giving a total of 48 replicates (N = 48) from a total of 3 donors and 5 eyes. The explants were placed in the center of the inserts covered with HAM, one explant per insert.

The cultures were incubated at 37°C with 5% CO_2_ in a medium consisting of HEPES-buffered DMEM containing sodium bicarbonate and Ham’s F12. The medium was supplemented with 5% fetal bovine serum, 0.5% dimethyl sulphoxide, 2 ng/mL human epidermal growth factor, 5 μg/mL insulin, 5 μg/mL transferrin, 5 ng/mL selenium, 3 ng/mL hydrocortisone, 30 ng/mL cholera toxin (Biomol, Exeter, UK), 50 μg/mL gentamycin, and 1.25 μg/mL amphotericin B [32] and changed every 2^nd^ to 3^rd^ day.

After 21 days of incubation, the experiment was terminated, and all the 48 cultures were stained with rhodamine to visualize the borders and photographed for examination of outgrowth area. Subsequently, samples from 6 LEC sheets cultured from large explants and 5 from small explants (all from donor 2) were taken with a 6 mm biopsy punch, fixed in 2% glutaraldehyde in 0.2 M cacodylate buffer adjusted to pH 7.4, for subsequent preparation for transmission electron microscopy. Finally, 15 of the cultures from the large explant group and 14 of the cultures from the small explant group (donors 2 and 3) were fixed in 4% formaldehyde for later preparation for Hematoxylin & Eosin (H&E) and immunohistochemical analyses.

**Fig 1:**
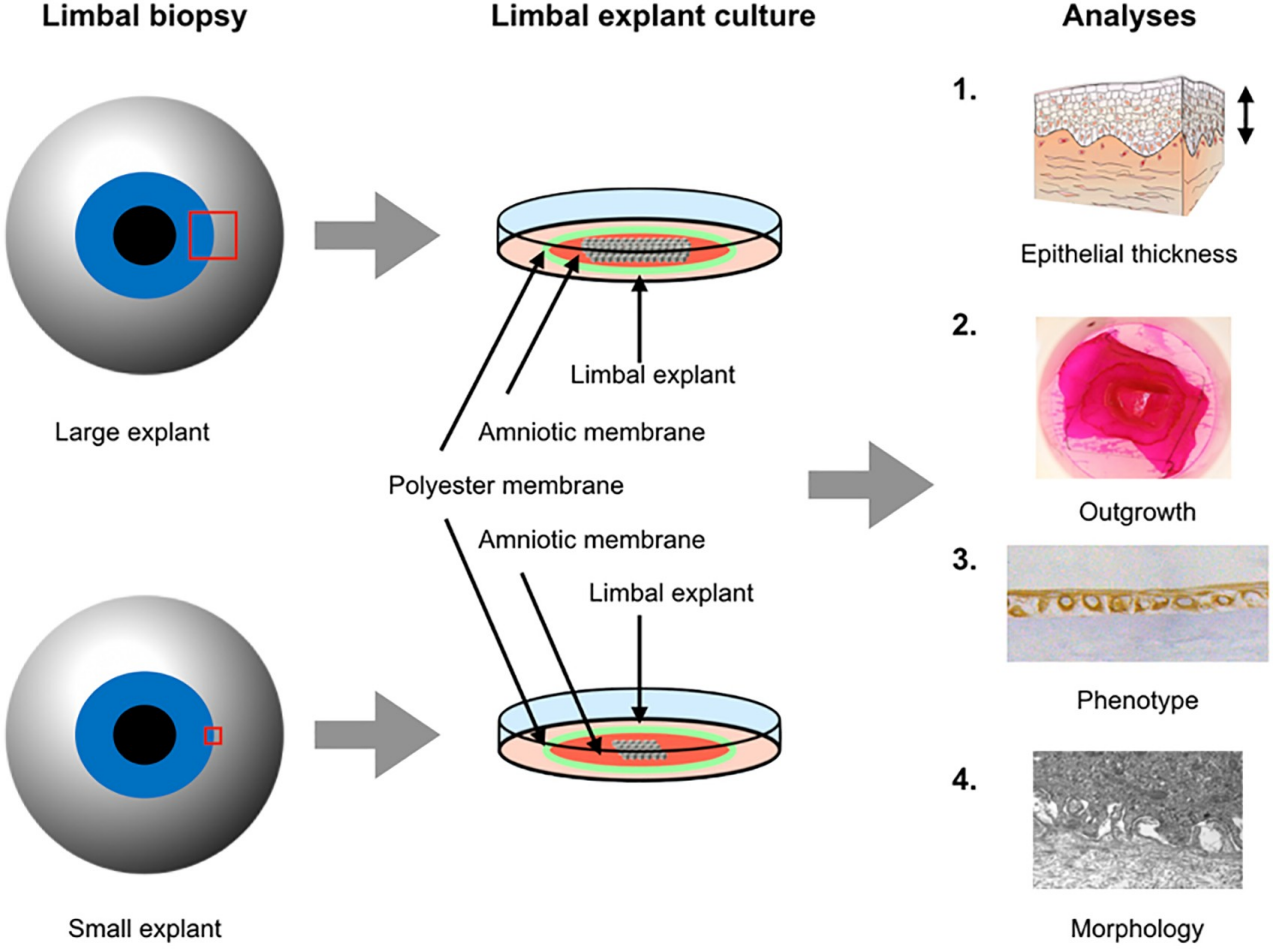
Experimental design of the study. Large (3 mm) and small (1 mm) limbal explants were cultured for three weeks on intact amniotic membranes fastened to polyester membranes of culture plate inserts for 3 weeks. Epithelial thickness and stratification, outgrowth, phenotype and morphology were compared between the groups.

For immunohistochemical analysis, the experiment behind the present study was initially designed to compare not only large versus small explants, but also explant orientation effect on phenotype, since a previous study by our research group found that a higher percentage of p63-positive, immature cells was achieved if the explants were oriented with the epithelial side facing the HAM [33]. Therefore, 7 cultures with the epithelial side of the explants facing the HAM and 8 cultures with the stromal side of the explants facing the HAM were distributed to the large explant group. Correspondingly, 6 cultures with the epithelial side of the explant side towards the HAM and 7 cultures with the stromal side of the explants facing the HAM was distributed to the small explant group, taking care of an even distribution of donors as well as explant orientation between the groups. We analyzed the two orientations separately and found no statistically significant differences for any of the immunohistochemical markers (Figs A, B, C, D, E, F, G, H, I, and J in S1 File, S1–S3 Tables), and therefore we decided to include all cultures regardless of explant orientation in the present large versus small explant study. The experimental design of the study is described in Fig 1.

### Histology and immunohistochemistry

Limbal tissue derived from large (3 mm, N = 15) and small (1 mm, N = 14) explants was fixed in neutral buffered 4% formaldehyde, dehydrated in increasing concentrations of ethanol, cleared with xylene and embedded in paraffin. Semithin (5 μm) cross-sections were stained with hematoxylin and eosin. For each sample, measurements of epithelial thickness and counting of cell layers was performed by two independent investigators, at 400x magnification and at regular intervals of 250 μm. Measurements were analyzed using Analysis software version 5 (Soft Imaging System GmbH, Muenster, Germany).

For immunohistochemical microscopy, serial 5 μm sections were immunostained with antibodies recognizing Keratin 19 (K19), ATP binding cassette transporter protein ABCG2, integrin β1, p63, p63α, Keratin 3 (K3), connexin 43 (Cx43), E-cadherin, Ki67 and PCNA (Table 1). Heat-induced epitope retrieval was performed. Thereafter, the sections were incubated with antibodies at 37°C overnight. Antibody diluent and a detection kit Ventana ultraView Universal DAB (760–500), an automated immunostaining system based on the ABC avidin-biotin-peroxidase method, were used (Ventana Medical Systems Inc. Tucson, AZ, USA). Both negative and positive controls were included. The expression of the various markers was determined at 400x magnification by two independent investigators and calculated as the number of positive cells/total number of cells x 100%.

### Assessment of epithelial outgrowth

After rhodamine B staining and photographing, ImageJ software (National Institutes of Health, Bethesda, MD) was used to mark the edges of the LEC sheets on the images and the areas were measured. The total area of the cultured LEC sheet including both the outgrowth area and the central explant area was included as outgrowth area. Fold growth in each culture was quantified as outgrowth size/explant size.

### Ultrastructure

LEC expanded from both small (N = 5) and large (N = 6) tissue samples were fixed in 2% glutaraldehyde in 0.2 M cacodylate buffer adjusted to pH 7.4, postfixed in 1% osmium tetroxide, and dehydrated through a graded series of ethanol up to 100%. The tissue blocks were then immersed twice in propylene oxide, for 20 minutes each, before being embedded in Epon (Electron Microsopy Sciences, Hatfield, PA, USA). Ultrathin sections were cut on a microtome (Leica Ultracut UCT; Leica, Wetzlar, Germany) and examined using a transmission electron microscope (TEM) (model CM120; Philips, Amsterdam, The Netherlands). TEM micrographs were taken from selected areas by an experienced technician blinded for the experimental groups. Desmosomes and hemidesmosomes per μm were counted by two independent investigators examining the micrographs.

### Statistical analysis

Statistical analysis was performed using IBM SPSS Statistics for Macintosh version 25 (IBM Corp, Armonk, NY). A significance level of 5% was used throughout the study. The independent T-test was used to compare means. Pearson’s correlation test was used to analyze the correlation between explant size, outgrowth size and fold growth. The data are presented as mean ± standard deviation (SD).

## Results

### Effect of explant size on stratification and epithelial thickness

**Fig 2:**
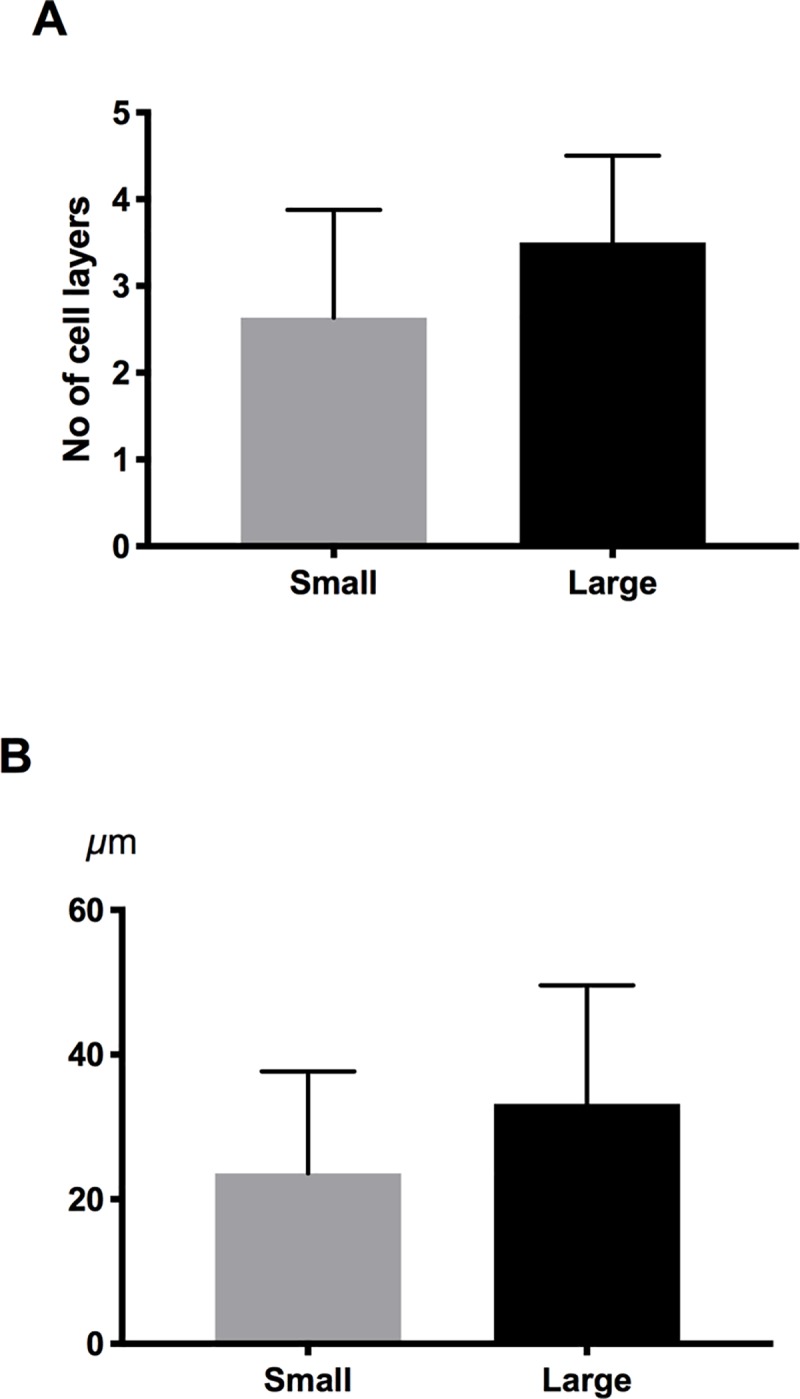
Epithelial thickness and stratification. Bar charts demonstrating the number of cell layers (A) and epithelial thickness (B) measured in LEC cultures derived from small and large explants. There were no significant differences between the groups. Error bars represent the standard deviation of mean values.

Stratification and thickness of cultured LEC was histologically determined by two independent investigators. The average number of cell layers in cultures derived from large (n = 13) and small (n = 11) explants was 2.6±1.2 and 3.5±1.0, respectively (P = 0.07) (Fig 2A, S4 Table). The average epithelial cell sheet thickness in cultures from large (n = 13) and small (n = 11) explants was 33.6±16.4 μm and 23.2±14.1 μm, respectively (P = 0.14) (Fig 2B, S4 Table). Explant size did not significantly affect stratification or thickness of the limbal epithelium.

### Effect of explant size on outgrowth

**Fig 3:**
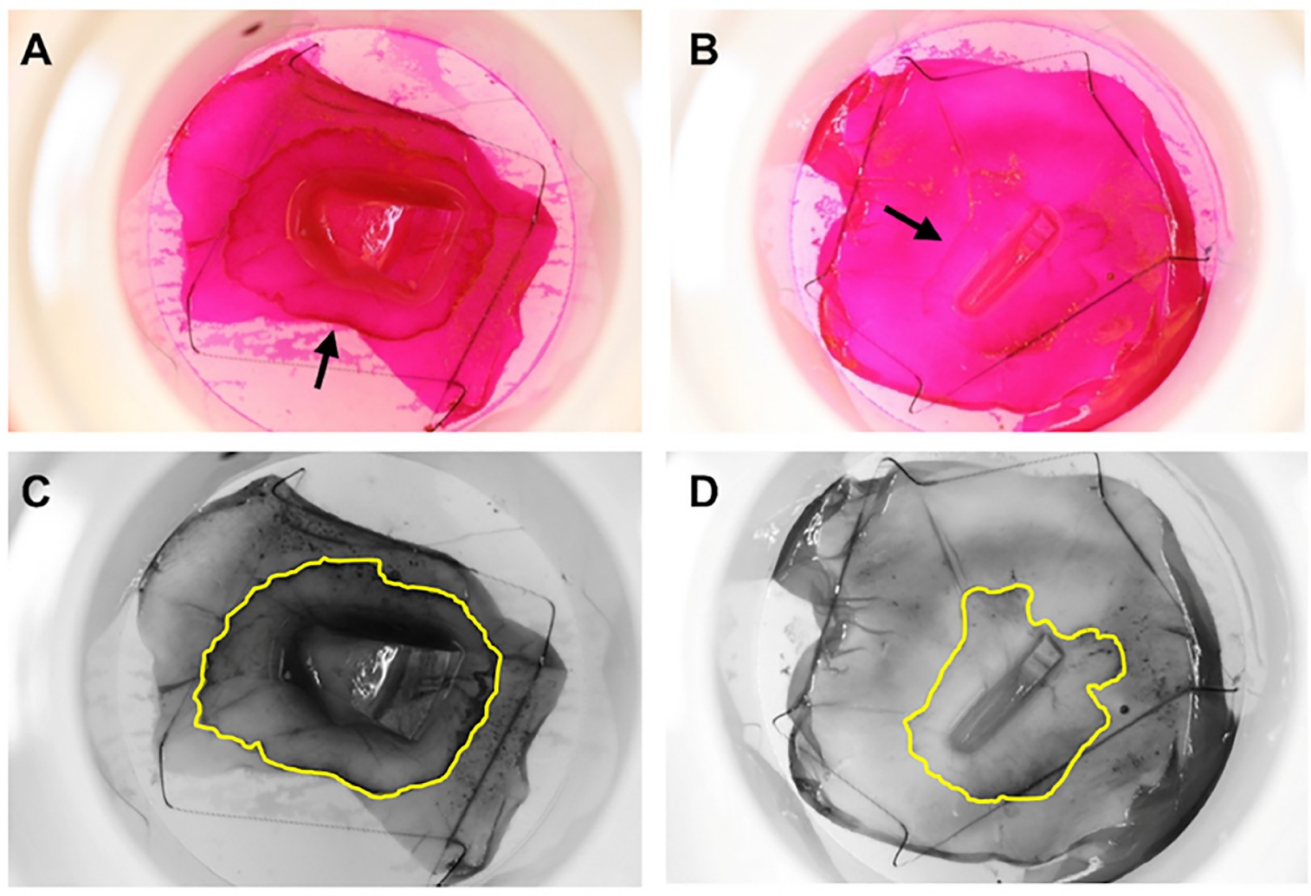
Rhodamine staining of small and large explants. Rhodamine staining of cultured limbal epithelial cells from large (A and C) and small (B and D) explants. A, and B are color photographs of the cultures, and C and D represent the same photographs processed by ImageJ. The corneoscleral explants can be seen in the center of the cultures. The leading edges of cultured limbal epithelial cells are indicated by arrows (A and B). The total areas of the cultured limbal epithelial sheets including the explant areas are highlighted in yellow (C and D).

**Fig 4:**
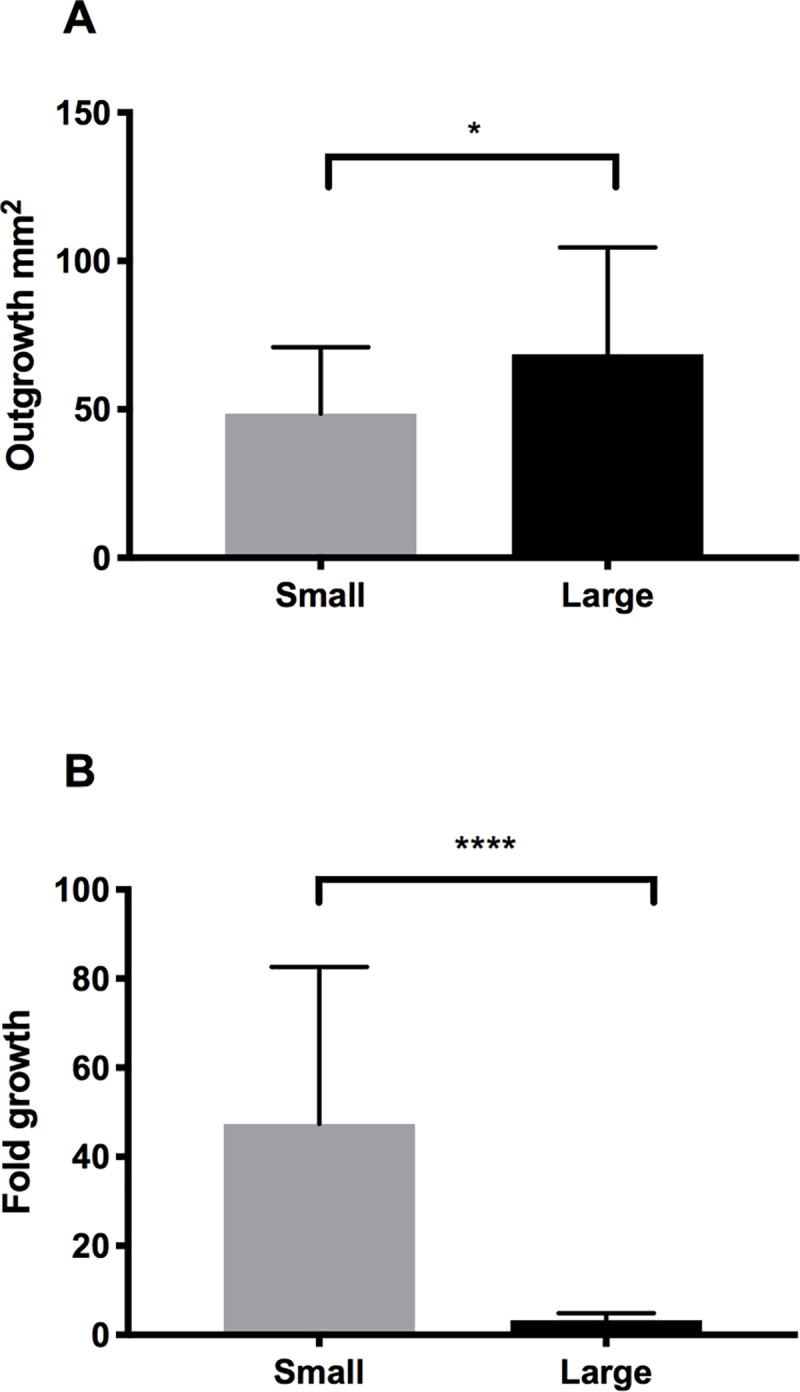
Outgrowth. Bar charts demonstrating the outgrowth (A) and fold growth (B) of LEC from small and large explants. *P < 0.01. Error bars represent the standard deviation of mean values.

Outgrowth of LEC cultures was assessed by rhodamine staining and quantified using ImageJ software (Fig 3). The large explants yielded significantly more outgrowth compared to the small explants (68.6±36.0 mm^2^ and 48.5±22.4 mm^2^, respectively (n = 48, *P* = 0.03) (Fig 4A, S5 Table). However, fold growth (outgrowth size/explant size) was significantly higher for the small explants (3.26±1.65. for large explants and 47.4±35.3 for small explants, respectively; N = 48, *P* < 0.0001) (Fig 4B, S5 Table). Outgrowth size correlated positively with explant size (*r* = 0.37; P = 0.01), whereas fold growth correlated negatively with explant size (*r* = –0.55; P < 0.0001). This indicates that small explants may have a higher growth potential and grow faster than large explants, yielding relatively larger outgrowth than large explants.

### Effect of explant size on desmosome and hemidesmosome densitY

**Fig 5:**
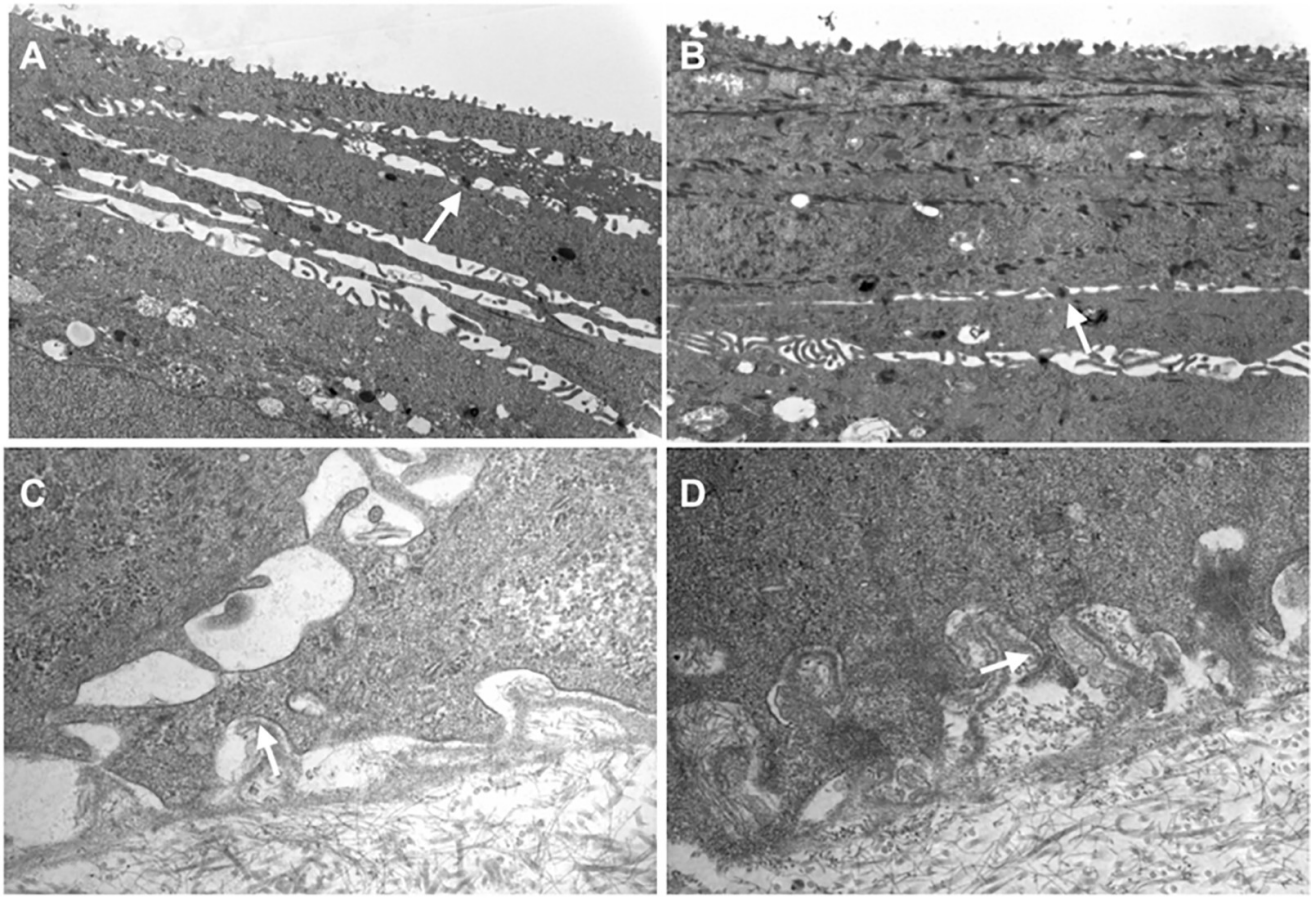
Ultrastructure of human LEC derived from small and large explants. Transmission electron microscopy showing desmosomes (A and B) and hemidesmosomes (C and D) of cultured cells derived from large (A and C) and small (B and D) human explants. White arrows indicate these structures.

**Fig 6:**
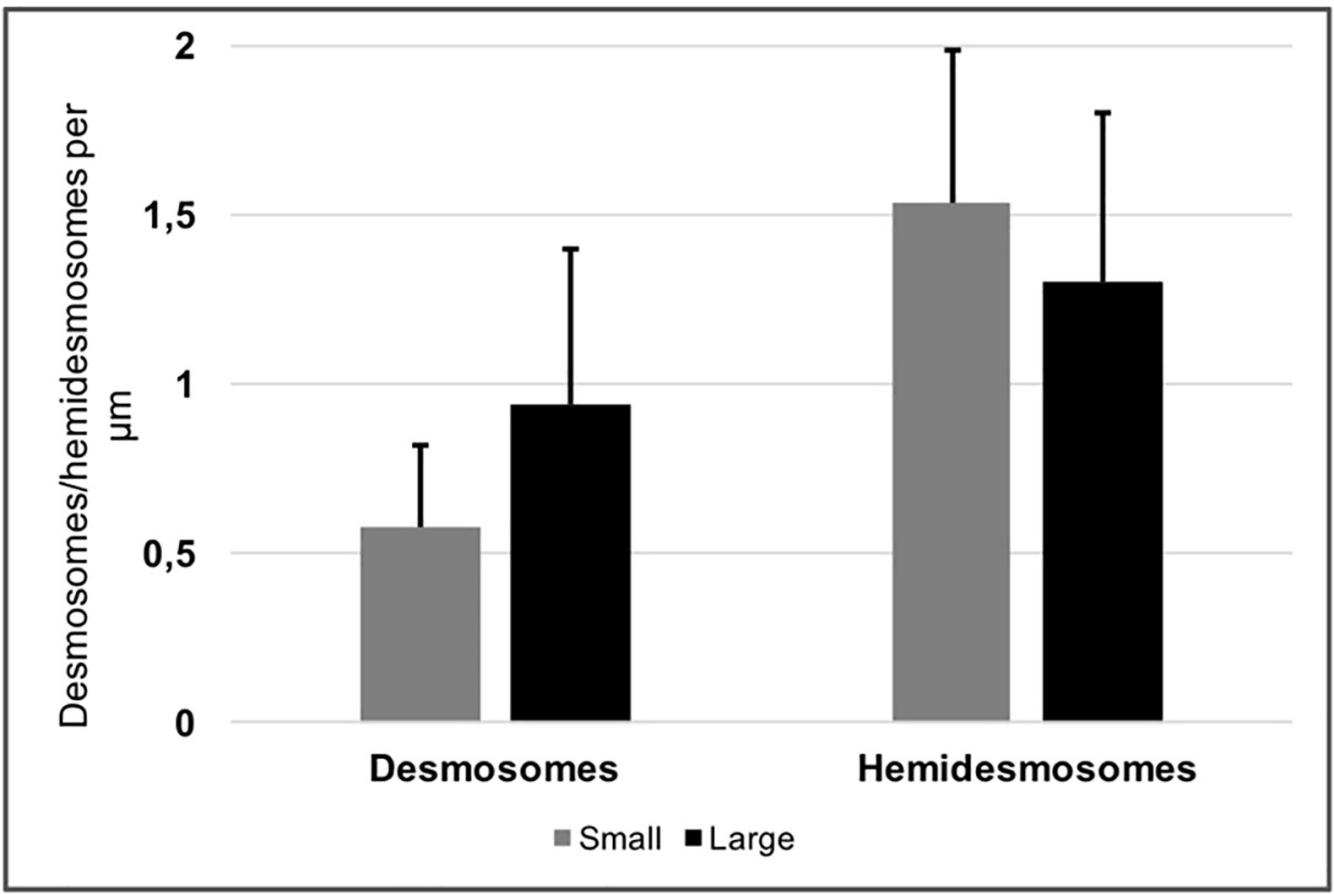
Quantification of desmosome and hemidesmosome density. Bar chart showing the number of desmosomes (black) and hemidesmosomes (grey) per μm in human LEC derived from small and large explants. There were no significant differences between the groups. Error bars represent the standard deviation of mean values.

Ultrastructure was evaluated by transmission electron microscopy, by counting the densities of desmosomes (Fig 5A and 5B, S6 Table) and hemidesmosomes (Fig 5C and 5D, S7 Table). Cultured cells derived from large explants had a total number of desmosomes per μm of 0.94±0.46 versus 0.57±0.24 for small explants (n = 11; *P* = 0.18) (Fig 6, S6 Table). The number of hemidesmosomes per μm was similar between cultured cells derived from large and small explants (1.3±0.50 compared to 1.54±0.45, respectively; (N = 11; *P* = 0.45) (Fig 6, S7 Table). These findings indicate that the structural integrity of LEC is maintained regardless of explant size.

### Effect of explant size on phenotype and proliferation

**Fig 7:**
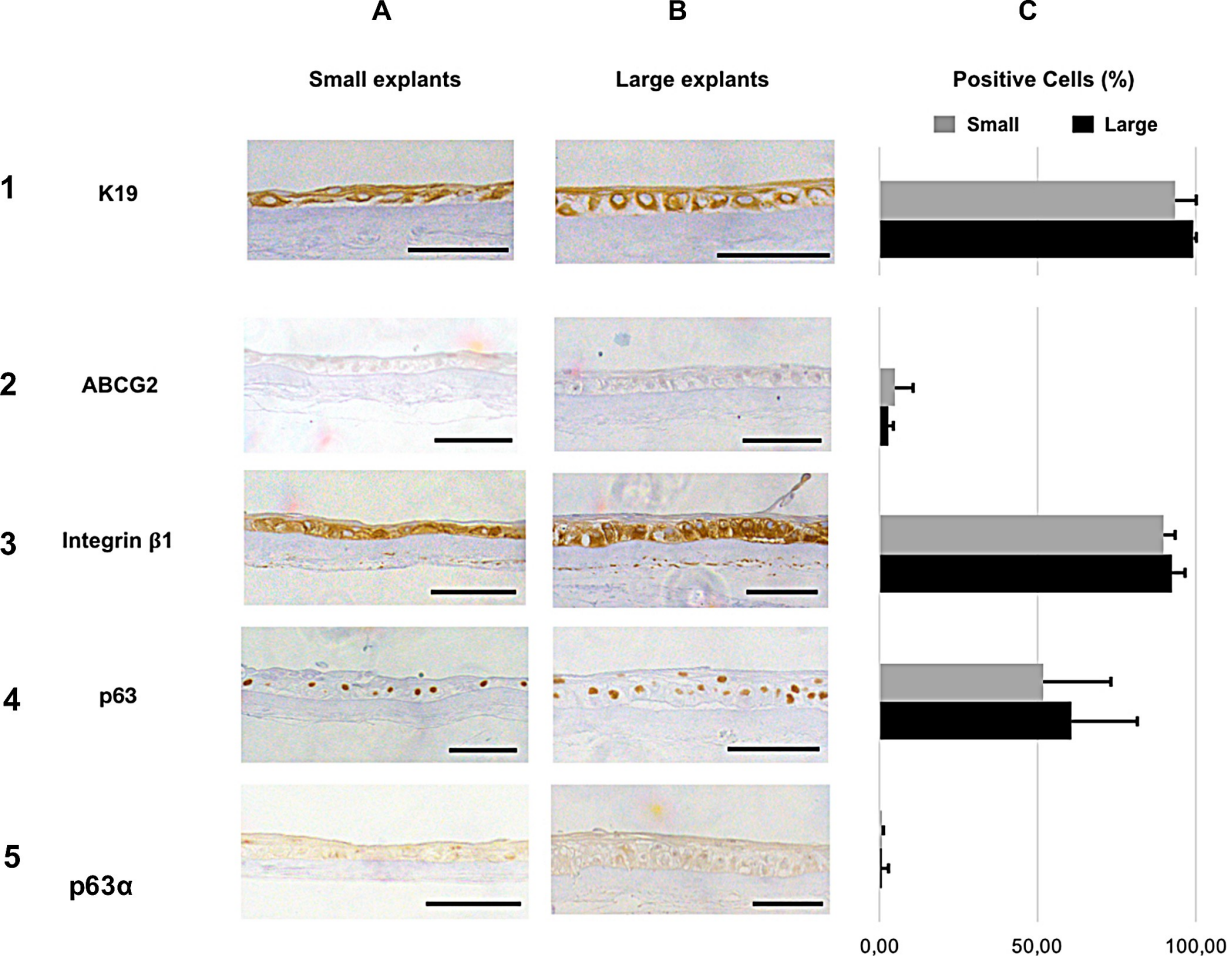
**Immunostaining (A and B) and Quantification (C) of immature phenotype markers. A and B.** Immunostaining of **1)** K19, **2)** ABCG2, **3)** integrin β1, **4)** p63, and **5)** p63α of cultured human LEC derived from small **(A)** and large **(B)** limbal explants. The selected areas in the micrographs are representative for the staining and not for the epithelial thickness in the study groups. Scale bar = 100 μm. **C**: Bar charts showing the percentage of positive cells for the immature phenotype markers in cultured LEC sheets cells derived from small (grey) and large (black) explants. *P < 0.05. **P < 0.01. Error bars represent the standard deviation of mean values.

The LEC marker K19 [34, 35] (Fig 7A and 7B, panel 1) was expressed in the cellular cytoplasm and was high in both groups, but significantly higher in the large explant group compared to the small group (99.1±1.2% and 93.2±13.6% of the cells, respectively; N = 28, *P* = 0.024, Fig 7C, panel 1, S2 and S3 Tables).

For all other immature cell markers, there were no significant differences in expression between large and small explant groups. ABCG2, a putative marker for limbal stem cells [36, 37] and their immediate progeny [38], (Fig 7A and 7B, panel 2, S2 and S3 Tables) was expressed in the cellular membranes at low levels for cultures from both large (2.6±1.9%) and small (5.0±5.4%) explants (N = 28, P = 0.14, Fig 7C, panel 2, S2 and S3 Tables). Integrin β1, a presumed progenitor LEC marker [39] (Fig 7A and 7B, panel 3, S2 and S3 Tables), was strongly expressed in cytoplasmic and cell membranes, with 92.3±4.4% of cells derived from large explants and 90.0±3.6% of cells derived from small explants (N = 28, P = 0.13, Fig 7C, panel 3, S2 and S3 Tables). The putative limbal stem cell/transit-amplifying cells marker p63 [40, 41], (Fig 7A and 7B, panel 4, S2 and S3 Tables), stained the nuclei of 60.6±20.7% of cells derived from large explants and 52.0±21.2% of cells derived from small explants (N = 28, P = 0.28, Fig 7C, panel 4, S2 and S3 Tables). The expression of the p63α isotypes (Fig 7A and 7B, panel 5) was barely detectable in the nuclei (0.8±1.9% of the cells in the large explant group and 0.4±0.9% of the cells in the small explant group) (n = 29, P = 0.51, Fig 7C, panel 5, S2 and S3 Tables), suggesting that the holoclone-associated, ΔNp63α fraction of the p63 positive cells was negative.

**Fig 8:**
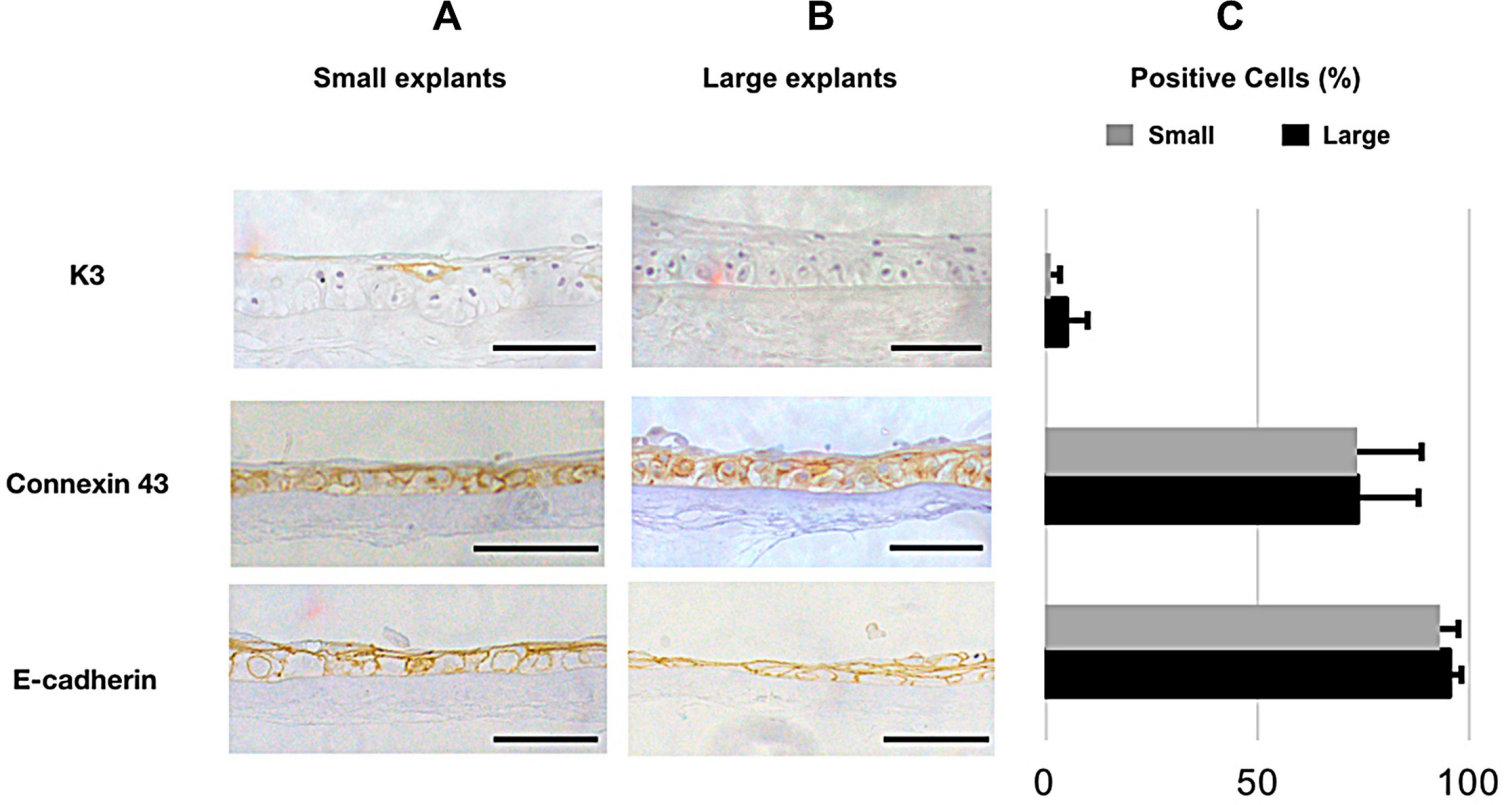
**Immunostaining (A and B) and Quantification (C) of differentiation markers. A, and B:** Immunostaining of **1)** K3, **2)** Connexin 43, and **3)** E-cadherin of cultured human LEC derived from small **(A)** and large **(B)** limbal explants. The selected areas in the micrographs are representative for the staining and not for the epithelial thickness in the study groups. Scale bar = 100 μm. **C:** Bar charts showing the percentage of positive cells for the differentiation markers in cultured LEC sheets cells derived from small (grey) and large (black) explants. Error bars represent the standard deviation of mean values.

Expression of the corneal differentiation marker K3 [42] (Fig 8A and 8B, panel 1, S2 and S3 Tables), was detected in the cytoplasm of clusters of cells in 0.6±1.9% of the cells in the small explant group and 4.6±5.7% in the large explant group (N = 29, *P* = 0.074, Fig 8C, panel 1, S2 and S3 Tables), suggesting an undifferentiated phenotype. In contrast, the percentage of the cells expressing the suggested negative stem cell marker Connexin 43 [43] (Fig 8A and 8B, panel 2, S2 and S3 Tables) was high and similar for both groups. A total of 73.4±14.7% of the cells from the large explant group and 73.5±15.2% of the cells in the small explant group stained positively for connexin 43 in the cell membranes (N = 26, P = 0.98, Fig 8C, panel 2, S2 and S3 Tables). Also for adherence junction marker E-cadherin that indicates differentiated type of cells[44, 45] (Fig 8A and 8B, panel 3, S2 and S3 Tables), staining of cell membranes were found in 95.4±2.7% cells in LEC derived from large explants and 93.1±4.6% for cells derived from small explants (N = 25, P = 0.13, Fig 8C, panel 3, S2 and S3 Tables).

**Fig 9:**
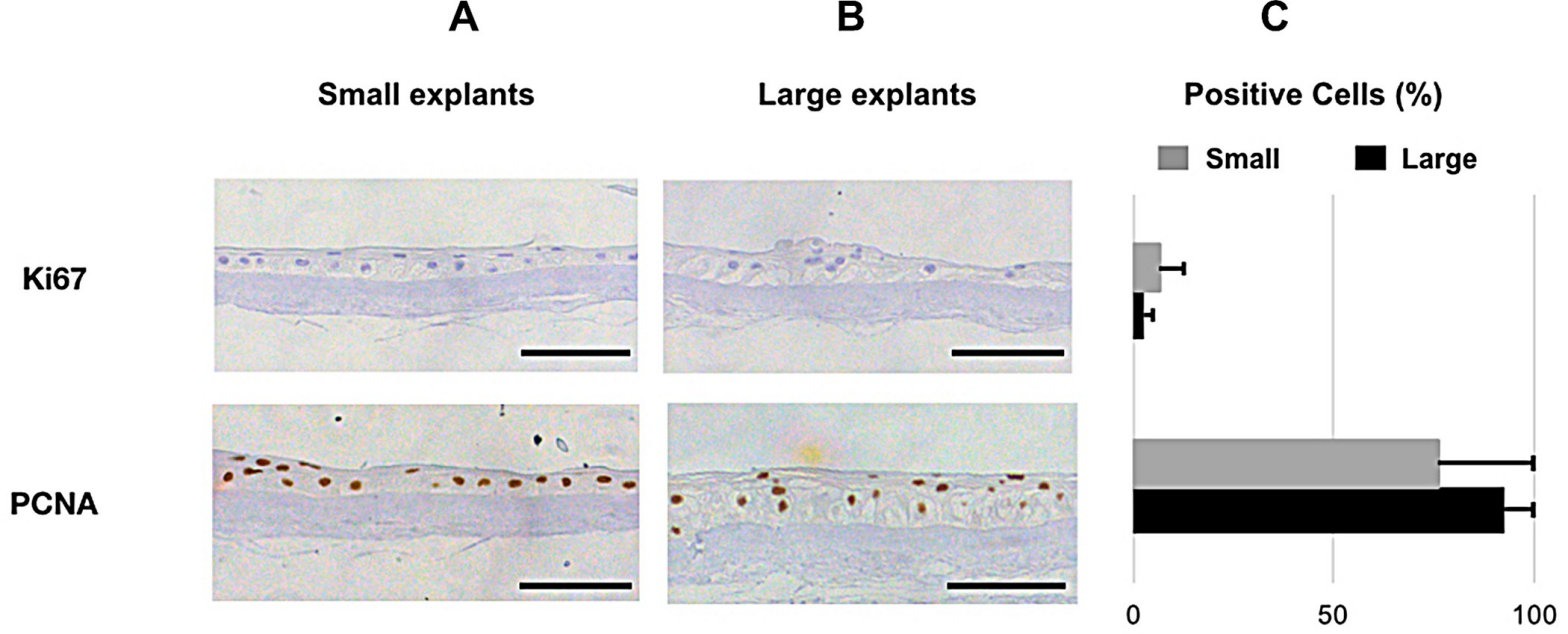
**Immunostaining (A and B) and Quantification (C) of proliferation markers. A, and B:** Immunostaining of **1)** Ki67, and **2)** PCNA of cultured human LEC derived from small **(A)** and large **(B)** limbal explants. The selected areas in the micrographs are representative for the staining and not for the epithelial thickness in the study groups. Scale bar = 100 μm. **C:** Bar charts showing the percentage of positive cells for the proliferation markers in cultured LEC sheets cells derived from small (grey) and large (black) explants. Error bars represent the standard deviation of mean values.

Two proliferation markers were considered, Ki67 [46] and PCNA [47]. Ki67 immunoreactivity was detected in the nuclei (Fig 9A and 9B, panel 1), and the number of cells expressing Ki67 was 2.7±2.0% in the large group compared to 6.3±6.2% of the cells in the small group (N = 27, *P* = 0.073, Fig 8C, panel 1, S2 and S3 Tables). PCNA staining was located in the nuclei (Fig 9A and 9B, panel 2, S2 and S3 Tables), and the number of cells stained was 92.8±15.3% in the large explant group and 76.3±38.6% in the small explant group (N = 14, P = 0.28, Fig 8C, panel 2, S2 and S3 Tables).

## Discussion

In the present study we investigated the effects of small versus large limbal explant size (1 mm versus 3 mm of the corneal circumference) on stratification, thickness, outgrowth area, fold growth, mechanical strength, phenotype and proliferation. We found that large explants were preferable to small in terms of outgrowth and preservation of an immature phenotype. However, the fold growth (outgrowth area /explant area) was higher in the small explant group and several quality parameters for transplants intended for limbal stem cell deficiency treatment (thickness, stratification, mechanical strength, p63 expression) were adequate for both the small and the large explant group.

Confluent, well-stratified LEC sheets with many cellular junctions were observed regardless of explant size. A graft with sufficient mechanical strength is preferable, as it reduces the risk of infection [48] and other severe post-operative complications [21] and is considered better equipped to resist the mechanical stress associated with transplantation.

The outgrowth capacity of limbal explants is of great clinical interest as these cells need to cover the anterior corneal surface, measuring approximately 132 mm^2^ [49]. The impressive outgrowth capacity of limbal explants was demonstrated by Kethiri *et al*. [25], who showed that explants as small as 0.3 mm^2^ for live and ≥ 0.5 mm^2^ for cadaveric tissue obtained an *in vitro* mean expansion area of 182 mm^2^ and 218 mm^2^, respectively. This is higher than the average outgrowth achieved in the present study. A possible explanation to the poorer outgrowth in the present study could be that all cultures were included regardless of growth capacity. In a clinical setting the cultures with insufficient growth would have been discarded. Some of the cultures in both groups yielded an outgrowth area above 100 mm^2^, while others turned out to be much smaller. The internal variation within the groups despite identical culture conditions suggests that factors other than the culture protocol determined the growth. For instance, limbal explants derived from the superior limbus have been shown to demonstrate the highest outgrowth [50], while the samples used in the present study were collected from all limbal regions. In addition, donor characteristics have proven to influence growth potential of LEC [39, 51–56]. These characteristics may vary between the studies.

In the present study, the total outgrowth area was higher in the large explant group, unlike what was reported by Kethiri *et al*. [25], where explants more than 0.3 mm^2^ for live donors and 0.5mm^2^ for cadaveric donors produced outgrowth areas of similar size. This could mean that in some cases where the growth is not sufficient due to unknown donor properties, the harvest of larger explants may be necessary. However, in the present study we also found that the fold growth (total outgrowth area divided with explant size) was higher in the small explant group. Based on these results, 3 mm explants could be divided in three pieces of 1 mm each, in order to increase the sum of outgrowth area yielded from the explant. It is likely that the beneficial effect of dividing the explants would be transferrable to even smaller pieces of tissue, down to a limit of e.g. 0.3 mm^2^ (live donors) or 0.5 mm^2^ (cadaveric donors) according to Kethiri *et al*. [25]. The finding that fold growth was higher for smaller explants is in line with Eidet *et al*.*’*s study of fold growth versus total growth of conjunctival biopsies [29]. As pointed out in Eidet *et al*. and studied by Li *et al*. and Komine *et al*. [57, 58], increased cytokine release from the damaged cells at the cut edge of the explant has been shown to propel cell outgrowth both in stromal fibroblasts and in keratinocytes. This is in agreement with Kolli *et al*., who found the amount of immature cells to decrease with the distance from the explants [23]. Also in skin biopsies, keratinocytes close to the cut edge have been shown to be more proliferative [58]. In summary, based on the present study, a limbal biopsy of at least 3 mm is preferable, but mincing of the biopsy prior to seeding would lead to better growth.

Perhaps the usage of multiple, minced explants could explain why SLET has proven so successful in terms of epithelial coverage and is not associated with the same amount of post-operative complications as other limbal transplantations without the culture of a covering epithelial sheet prior to surgery [16]. Also, as a result of the present findings and in light of previous literature [23, 25], a possible improvement of the explant technique would be to divide explants immediately prior to culture, and seed more than one explant onto the same carrier.

The potential mechanical strength of the cell layer was determined indirectly through assessment of desmosome and hemidesmosome density, which is known to be high in limbal stem cells [59]. The LEC sheets in the present study appeared well attached, and desmosome/hemidesmosome density reported herein was higher for both small and large explant groups than in a previous study by our research group [33]. Hence, the use of small versus large explants for culture does not seem to affect the mechanical strength of the LEC sheets.

The identification of putative limbal stem cells has been based on the cells expressing putative stem cell/progenitor cell markers and correspondingly lack of differentiation marker expression. In the present study, limbal epithelial sheets from both small (1 mm) and large (3 mm) explants after 21 days of culture were similar in terms of expression of both putative stem cell/progenitor cell markers, differentiation markers and proliferation markers, apart from K19, where the percentage of cells staining positive for K19 was significantly higher in the large limbal explant group compared to the small explant group. K19 was suggested to be a keratinocyte stem cell marker [60] and a putative limbal stem cell marker specific for basal LEC [34]. K19 expression was later proven to be abundant in both suprabasal and basal LEC and conjunctival epithelial cells [35], while its expression in cornea was weak and located to basal epithelial cells in the periphery [35]. Hence, K19 marks limbal epithelial cells as opposed to corneal epithelial cells. The clinical significance of the higher percentage of K19-positive cells in the large explant group than for the small explant group is uncertain.

ABCG2 was expressed at low levels without significant differences between groups. ABCG2 is in a number of different organ systems where it was proposed to be a marker of a side population of cells that contain, but not exclusively represent, stem cells [61–65]. In the ocular surface, ABCG2 is primarily expressed in clusters in limbal and conjunctival basal cells [66, 67] and was proposed as a putative marker of limbal stem cells [36, 37] and their immediate progeny [38]. In Kethiri *et al*. cultured LEC sheets from cadaveric and living donor explants of varying sizes were compared, and ABCG2-expressing cells were found nearer to the edges of the expansion area [25]. It was not specified whether ABCG2-staining varied with different explant sizes. A similar localization with cells staining positive for ABCG2 at the culture edges was not observed in the present study. The low level of positive staining for ABCG2 in the present study is in contrast to an earlier study from our group that detected a very high percentage of cells staining positive for ABCG2. Similar culture methods were used for both studies except for the culture time that was 21 days in the present study and 14 days in the other study [68]. Possibly, the longer culture time may have shifted the cells towards a more differentiated phenotype.

Integrin β1 is a proposed keratinocyte stem cell marker and has been shown to be expressed in small, basal limbal cells that also stain positive for the putative limbal stem cell/progenitor/transit-amplifying cell marker P63 [39, 66, 69]. In the present study, integrin β1 was abundant and equally expressed in LEC derived from both small and large explants. In an earlier study by our group, this marker was expressed at low levels [70]. In comparison, Kim *et al*. [39], performed a study where 26% of cells cultured from limbal explants were integrin β1 positive. Their culture protocol was similar to the present study, except that explants were not subjected to dispase prior to seeding but were covered with drops of FBS and left overnight to secure attachment to the substrate. Integrin β1 is a transmembrane protein involved in cell-matrix adhesion and signaling between the cell and extracellular matrix [69]. Possibly, the difference in initial method for cell adhesion between Kim *et al*. [39] and the present study may have influenced the final percentage of cells expressing integrin β1.

For limbal tissue, a level of >3% p63 brightly positive cells is correlated with treatment success [71]. Herein, the transcription factor p63 was expressed by 60% of cells derived from large explants and 52% of cells derived from small explants. The similar moderate to high expression of p63 in both our limbal explant groups suggests that both small and large explants may yield LEC sheets with a suitable phenotype for restoration of the corneal epithelium. The nuclear expression of p63α, which also includes the ΔNp63α isotype that is known to mark holoclones [41, 72], was negative in both groups in the present study. This finding suggests that the majority of p63 positive cells represented transit-amplifying cells and not limbal stem cells. This is in contrast with a study by Eidet *et al*. [29] of epithelia cultured from conjunctival biopsies of different sizes. Eidet *et al*. [29] found that the expression of the p63 marker and proliferation marker PCNA were increased in the large conjunctival biopsy group [29]. In Kethiri *et al*. when cultured LEC sheets from cadaveric and living donor explants of varying sizes were compared, and cells positive for ΔNp63α was dispersed across the cultures [25]. Kethiri et al. cultured the explants for 8 days in contrast to 21 days in the present study. The long culture time may have depleted the LEC sheets for p63α-expressing cells.

Three suggested differentiation-associated markers (K3, Connexin 43, and E-cadherin) were evaluated. In the ocular surface, K3 is a marker of terminally differentiated corneal epithelial cells [42]. The limbal basal epithelium, containing the limbal epithelial stem cell population, does not express K3. However, the marker is present when the limbal stem cell differentiates into the corneal epithelium [42]. In our study, the percentage of cells staining positively for K3 was low, reflecting an immature phenotype. Occasionally, clusters of cells positive for K3 were observed within the LEC sheets, demonstrating a differentiation into corneal type of epithelium. The percentage of cells positive for K3 tended to be lower in the large explant group. Kethiri *et al*. also found that K3 was expressed in cultures from both large and small explants from cadaveric and live donors, but variations in expression between explants of different sizes were not investigated [25].

Connexin 43 is a gap junction protein that is considered as a differentiation marker because of its absence in the limbal basal epithelium and presence in the corneal basal epithelium [73]. More specifically, it denotes differentiation of limbal stem cells to transient amplifying cells [74]. The marker was equally expressed in both culture groups, at moderate to high percentages. A high level of connexin 43 is in line with earlier findings by our group [68].

Percentages of cells positive for E-cadherin were high in both the small and large explant groups. E-cadherin is a type of transmembrane protein that takes part in adherence junctions and provides strong mechanical attachments for neighboring epithelial cells [44, 45]. It is essential for directional migration of epithelial sheets and mediates healing of epithelial wounds [44]. Hence, a strong and abundant staining for E-cadherin in LEC for both groups in the present study is in concordance with the well attached LEC sheets and presence of desmosomes in the TEM analysis. Moderate levels of cells staining positive for E-cadherin was also found in an earlier study that used the same explant culture protocol as in the present study [70]. E-cadherin staining was also present in LEC sheets cultured from explants of varying sizes in Kethiri *et al*. [25].

Cell proliferation was assessed using PCNA and Ki67 staining. PCNA has a half-life of more than 20 hours and therefore may also be detected in resting (G_0_-phase) cells [47]. Ki67 is a nuclear antigen present in proliferating cells, but absent in resting cells [46]. Hence, Ki67 is a more specific proliferation marker. The percentage of cells staining positive for PCNA was high in both groups. Ki67-staining was Ki67 was 2.7±2.0% in the large group compared to 6.3±6.2% of the cells in the small group (N = 27, *P* = 0.073low in both groups compared to previous studies [70, 75], 2.7±2.0% in the large group versus 6.3±6.2% in the small group (*P* = 0.073). Eidet et al. found that explant size and outgrowth size were positively correlated with PCNA, suggesting higher proliferation in large explant cultures of conjunctival tissue [29], in contrast to the present study. Another group described progressive loss of cells staining positive for Ki67 with increasing culture duration, and very few Ki67 positive cells after a three-week culture period like the one employed herein [76]. A culture time of 21 days may have caused a decline in proliferation in the graft, which may be unfortunate prior to transplantation.

We could not find any difference in differentiation of cultures from large versus small explants based on the putative stem cell/progenitor cell markers and differentiation markers used. However, another recent marker, ATP-binding cassette, sub-family B, member 5 (ABCB5) has been shown to specifically identify mammalian limbal stem cells with the ability to restore the corneal epithelium upon grafting to limbal stem cell-deficient mice [77]. ABCB5 staining is not yet performed in clinical studies of limbal stem cell culture and transplantation.

In summary, for the present donor- and culture conditions, the harvesting of a large (3 mm) explant seems to be preferable to a small (1 mm) in terms of outgrowth area. As regards to LEC thickness, stratification, mechanical strength, and the attainment of a predominantly immature phenotype, both large and small explants are sufficient. Mincing the explant prior to culture and seeding of multiple limbal pieces onto the substrate could increase the yield of outgrowth area and may be an improvement to the existing protocols of ex vivo expansion of LEC with the explant technique.

## Supporting information

S1 File Figures A, B, C, D, E, F, G, H, I, and J. **Quantification of Immunohistochemcial markers for cultures with regards to explant orientation. A**. Keratin 19, **B**. ABCG2, **C**. Integrin β1, **D**. p63, **E**. p63α, **F**. Keratin 3, **G**. Connexin 43, **H**. E-cadherin, **I**. Ki67, **J**. PCNA. Epithelial group: Explants were oriented with the epithelium facing the intact amniotic membrane. Stromal group: Explants were oriented with the stroma facing the intact amniotic membrane. Error bars = + 1 Standard Deviation. The numbers are positively stained cells as a fraction of total number of cells /sample (1.00 = 100%).
(DOCX)

S1 Table Descriptives and t-test results for marker positivity of the cultures with regards to explant orientation. Epithelial group. Explants were oriented with the epithelium facing the intact amniotic membrane. Stromal group. Explants were oriented with the stroma facing the intact amniotic membrane. The numbers are positively stained cells as a fraction of total number of cells /sample (1.00 = 100%).
(DOCX)

S2 Table Immunohistochemical data for large (3mm) explant samples. Fraction of cells staining positive for the respective markers (1.00 = 100%). Sections with less than 100 cells were excluded from the dataset. Different letters of the samples mean different donors. Capital letters represent eyes in which the explants are oriented with the stroma facing the intact amniotic membrane (stromal group, grey background). Undercase letters represent samples where explants are oriented with the epithelium facing the intact amniotic membrane (epithelial group, white background).
(DOCX)

S3 Table Immunohistochemical data for small (1mm) explant samples. Fraction of cells staining positive for the respective markers (1.00 = 100%). Sections with less than 100 cells were excluded from the dataset. Different letters of the samples mean different donors. Capital letters represent eyes in which the explants are oriented with the stroma facing the intact amniotic membrane (stromal group, grey background). Undercase letters represent samples where explants are oriented with the epithelium facing the intact amniotic membrane (epithelial group, white background).
(DOCX)

S4 Table Mean thicknesses and mean numbers of cell layers per sample based on histologic sections. Sample names with uneven numbers (grey background) represent large (3 mm) explants. Even numbers (white background) mean small (1 mm) explants.
(DOCX)

S5 Table ImageJ area measurements based on Rhodamine stained culture images. Sample names with uneven numbers (grey background) represent large (3 mm) explants. Even numbers (white background) mean small (1 mm) explants.
(PDF)

S6 Table Desmosomes per length based on transmission electron microscopy micrographs. Sample names with uneven numbers (grey background) represent large (3 mm) explants. Even numbers (white background) mean small (1 mm) explants.
(DOCX)

S7 Table Hemi- desmosomes per length based on transmission electron microscopy micrographs. Sample names with uneven numbers (grey background) represent large (3 mm) explants. Even numbers mean small (1 mm) explants.
(DOCX)
