# Supplementary material for: Effects of explant size on epithelial outgrowth, thickness, stratification, ultrastructure and phenotype of cultured limbal epithelial cells
Source: PLoS One. 2019 Mar 12;14(3):e0212524. doi: 10.1371/journal.pone.0212524 (PMC6413940; doi:10.1371/journal.pone.0212524)
Supplement: S1 File — Quantification of Immunohistochemcial markers for cultures with regards to explant orientation. A. Keratin 19, B. ABCG2, C. Integrin β1, D. p63, E. p63α, F. Keratin 3, G. Connexin 43, H. E-cadherin, I. Ki67, J. PCNA. Epithelial group: Explants were oriented with the epithelium facing the intact amniotic membrane. Stromal group: Explants were oriented with the stroma facing the intact amniotic membrane. Error bars = + 1 Standard Deviation. The numbers are positively stained cells as a fraction of total number of cells /sample (1.00 = 100%). (DOCX) [file pone.0212524.s001.docx]

# S1 File. **Figures A, B, C, D, E, F, G, H, I, and J. Quantification of Immunohistochemcial markers for cultures with regards to explant orientation.**

# Keratin 19, **B**. ABCG2, **C**. Integrin β1, **D**. p63, **E**. p63α, **F**. Keratin 3, **G**. Connexin 43, **H**. E-cadherin, **I**. Ki67, **J**. PCNA.

**Epithelial group.** Explants were oriented with the epithelium facing the intact amniotic membrane**.**

**Stromal group.** Explants were oriented with the stroma facing the intact amniotic membrane.

**Error bars** = + 1 Standard Deviation

The numbers are positively stained cells as a fraction of total number of cells /sample (1.00 =100%).

**A**. Keratin 19, **B**. ABCG2, **C**. Integrin β1, **D**. p63, **E**. p63α, **F**. Keratin 3, **G**. Connexin 43, **H**. E-cadherin, **I**. Ki67, **J**. PCNA.

**A. B. C. D.**

**E. F. G. H.**

**I. J.**
