# Supplementary material for: Effects of explant size on epithelial outgrowth, thickness, stratification, ultrastructure and phenotype of cultured limbal epithelial cells
Source: PLoS One. 2019 Mar 12;14(3):e0212524. doi: 10.1371/journal.pone.0212524 (PMC6413940; doi:10.1371/journal.pone.0212524)
Supplement: S1 Table — Epithelial group. Explants were oriented with the epithelium facing the intact amniotic membrane. Stromal group. Explants were oriented with the stroma facing the intact amniotic membrane. The numbers are positively stained cells as a fraction of total number of cells /sample (1.00 = 100%). (DOCX) [file pone.0212524.s002.docx]

# S1 Table. Descriptives and t-test results for marker positivity of the cultures with regards to explant orientation

**Epithelial group.** Explants were oriented with the epithelium facing the intact amniotic membrane**.**

**Stromal group.** Explants were oriented with the stroma facing the intact amniotic membrane.

The numbers are positively stained cells as a fraction of total number of cells /sample (1.00 =100%)
