# Supplementary material for: Effects of explant size on epithelial outgrowth, thickness, stratification, ultrastructure and phenotype of cultured limbal epithelial cells
Source: PLoS One. 2019 Mar 12;14(3):e0212524. doi: 10.1371/journal.pone.0212524 (PMC6413940; doi:10.1371/journal.pone.0212524)
Supplement: S3 Table — Fraction of cells staining positive for the respective markers (1.00 = 100%). Sections with less than 100 cells were excluded from the dataset. Different letters of the samples mean different donors. Capital letters represent eyes in which the explants are oriented with the stroma facing the intact amniotic membrane (stromal group, grey background). Undercase letters represent samples where explants are oriented with the epithelium facing the intact amniotic membrane (epithelial group, white background). (DOCX) [file pone.0212524.s004.docx]

# S3 Table. Immunohistochemical data for small (1mm) explant samples

Fraction of cells staining positive for the respective markers (1.00 = 100%). Sections with less than 100 cells were excluded from the dataset. Different letters of the samples mean different donors. Capital letters represent eyes in which the explants are oriented with the stroma facing the intact amniotic membrane (stromal group, grey background). Undercase letters represent samples where explants are oriented with the epithelium facing the intact amniotic membrane (epithelial group, white background).

| Sample | ABCG2 | p63 | dNp63 | K19 | K3 | PCNA | Cx43 | Ki 67 | E-cadherin | β1 Integrin |
| --- | --- | --- | --- | --- | --- | --- | --- | --- | --- | --- |
| B2 | 0.11 |  | 0.03 | 0.99 | 0.015 | 0.975 | 0.81 | 0.025 | 0.96 | 0.885 |
| B4 | 0.05 | 0.71 | 0.00 | 0.97 | 0.00 | 0.86 | 0.65 | 0.00 | 0.96 | 0.935 |
| B6 | 0.04 | 0.675 | 0.005 | 0.97 | 0.05 |  | 0.805 | 0.02 | 0.85 | 0.87 |
| B8 | 0.05 |  | 0.02 | 0.985 | 0.015 | 0.99 | 0.445 | 0.05 | 0.95 | 0.845 |
| B10 | 0.055 | 0.59 | 0.00 | 0.98 | 0.055 | 0.955 | 0.47 |  | 0.87 | 0.9 |
| b2 | 0.03 | 0.77 | 0.00 | 0.91 | 0.005 | 0.93 | 0.775 | 0.03 | 0.955 | 0.935 |
| b4 | 0.00 |  | 0.00 |  | 0.00 |  |  |  |  | 0.895 |
| b6 | 0.06 | 0.645 | 0.00 | 0.985 | 0.025 | 0.3 | 0.78 | 0.015 |  | 0.87 |
| b8 | 0.035 | 0.645 | 0.005 | 0.995 | 0.01 | 0.975 | 0.775 | 0.07 | 0.945 | 0.89 |
| b10 | 0.02 | 0.065 | 0.00 | 1.00 | 0.005 | 1.00 | 0.955 | 0.045 | 0.885 | 0.98 |
| C2 | 0.21 | 0.32 | 0.00 | 1.00 | 0.04 | 0.975 | 0.675 | 0.04 |  | 0.92 |
| C4 | 0.015 | 0.64 | 0.00 | 0.5 | 0.01 | 0.995 | 0.805 | 0.2 | 0.965 |  |
| C6 | 0.00 | 0.185 | 0.00 |  | 0.00 |  |  | 0.095 | 0.965 | 0.87 |
| C8 | 0.02 | 0.54 | 0.00 | 0.98 | 0.00 | 0.96 | 0.88 | 0.165 | 0.985 | 0.885 |
