# Supplementary material for: Effects of explant size on epithelial outgrowth, thickness, stratification, ultrastructure and phenotype of cultured limbal epithelial cells
Source: PLoS One. 2019 Mar 12;14(3):e0212524. doi: 10.1371/journal.pone.0212524 (PMC6413940; doi:10.1371/journal.pone.0212524)
Supplement: S4 Table — Sample names with uneven numbers (grey background) represent large (3 mm) explants. Even numbers (white background) mean small (1 mm) explants. (DOCX) [file pone.0212524.s005.docx]

# S4 Table. Mean thicknesses and mean numbers of cell layers per sample based on histologic sections

Sample names with uneven numbers (grey background) represent large (3 mm) explants. Even numbers (white background) mean small (1 mm) explants.

| Sample | Mean thickness in µm | No of layers |
| --- | --- | --- |
| 1 - b1 | 44 | 4 |
| 1 - b2 | 20 | 3 |
| 1 - b3 | 35 | 4 |
| 1 - b5 | 36 | 4 |
| 14 - b6 | 23 | 3 |
| 14 - b7 | 17 | 2 |
| 14 - b8 | 59 | 6 |
| 14 - b9 | 78 | 6 |
| 14 - b10 | 27 | 3 |
| 27 - B1 | 48 | 5 |
| 27 - B2 | 20 | 2 |
| 27 - B3 | 14 | 3 |
| 24 - B5 | 23 | 3 |
| 40 - B6 | 9 | 1 |
| 40 - B7 | 25 | 3 |
| 40 - B8 | 15 | 2 |
| 40 - B9 | 30 | 3 |
| 40 - B10 | 35 | 4 |
| 53 - C2 | 17 | 3 |
| 53 - C3 | 25 | 3 |
| 53 - C4 | 8 | 1 |
| 66 - C7 | 28 | 3 |
| 66 - C8 | 25 | 3 |
| 66 - C9 | 29 | 3 |
