# Supplementary material for: Effects of explant size on epithelial outgrowth, thickness, stratification, ultrastructure and phenotype of cultured limbal epithelial cells
Source: PLoS One. 2019 Mar 12;14(3):e0212524. doi: 10.1371/journal.pone.0212524 (PMC6413940; doi:10.1371/journal.pone.0212524)
Supplement: S5 Table — Sample names with uneven numbers (grey background) represent large (3 mm) explants. Even numbers (white background) mean small (1 mm) explants. (PDF) [file pone.0212524.s006.pdf]

S5 Table. ImageJ area measurements based on Rhodamine stained culture images

Sample names with uneven numbers (grey background) represent large (3 mm) explants. Even numbers (white background) mean small (1 mm) explants.

Sample	**Outgrowth area (square mms)**	**Explant area (square mms)**
DA-H1	52.677	15.155
DA-H2	74.073	6.149
DA-H3	47.707	22.974
DA-H4	40.443	6.530
DA-H5	102.956	20.638
DA-H6	60.501	10.893
DA-H7	57.642	23.411
DA-H8	25.731	11.130
DA-V1	57.455	23.547
DA-V2	43.893	11.431
DA-V3	34.583	18.908
DA-V4	58.932	6.541
DA-V5	31.960	17.161
DA-V6	13.399	5.459
DA-V7	93.854	23.290
DA-V8	68.706	6.854
DB-H1	121.525	17.538
DB-H2	28.057	7.887
DB-H3	32.910	16.679
DB-H4	23.162	6.504
DB-H5	41.491	18.542
DB-H6	57.501	11.363
DB-H7	36.657	18.235
DB-H8	104.148	7.710

Sample	**Outgrowth area (square mms)**	**Explant area (square mms)**
DB-V1	52.187	14.427
DB-V2	42.655	11.998
DB-V3	53.878	20.270
Db-V4	86.992	6.229
DB-V5	66.364	24.021
DB-V6	39.880	6.061
DB-V7	60.666	17.693
DB-V8	64.829	7.187
DC-H1	62.075	24.106
DC-H2	62.513	9.012
DC-H3	43.738	24.853
DC-H4	19.213	4.409
DC-H5	45.180	24.735
Dc-H6	37.123	10.157
DC-H7	45.929	28.422
DC-H8	27.480	8.901
DC-V1	81.460	23.305
DC-V2	30.186	6.408
DC-V3	128.676	20.324
DC-V4	39.930	7.180
DC-V5	137.463	29.075
DC-V6	51.967	7.397
DC-V7	157.746	22.154
DC-V8	63.620	5.805
