# Supplementary material for: Effects of explant size on epithelial outgrowth, thickness, stratification, ultrastructure and phenotype of cultured limbal epithelial cells
Source: PLoS One. 2019 Mar 12;14(3):e0212524. doi: 10.1371/journal.pone.0212524 (PMC6413940; doi:10.1371/journal.pone.0212524)
Supplement: S6 Table — Sample names with uneven numbers (grey background) represent large (3 mm) explants. Even numbers (white background) mean small (1 mm) explants. (DOCX) [file pone.0212524.s007.docx]

# S6 Table. Desmosomes per length based on Transmission electron microscopy micrographs

Sample names with uneven numbers (grey background) represent large (3 mm) explants. Even numbers (white background) mean small (1 mm) explants.

| Sample | Length(µm) | Number of desmosomes | Desmosomes/µm |
| --- | --- | --- | --- |
| b1 | 82.85 | 42 | 0.51 |
| b2 | 84.67 | 32 | 0.38 |
| b3 | 82.16 | 63 | 0.77 |
| b5 | 124.03 | 99 | 0.80 |
| b6 | 104.56 | 91 | 0.87 |
| B1 | 81.48 | 152 | 1.87 |
| B2 | 94.08 | 63 | 0.67 |
| B3 | 82.73 | 47 | 0.57 |
| B5 | 88.54 | 104 | 1.17 |
| B6 | 80.51 | 31 | 0.39 |
| B7 | 86.07 | 79 | 0.92 |
