# Supplementary material for: Effects of explant size on epithelial outgrowth, thickness, stratification, ultrastructure and phenotype of cultured limbal epithelial cells
Source: PLoS One. 2019 Mar 12;14(3):e0212524. doi: 10.1371/journal.pone.0212524 (PMC6413940; doi:10.1371/journal.pone.0212524)
Supplement: S7 Table — Sample names with uneven numbers (grey background) represent large (3 mm) explants. Even numbers mean small (1 mm) explants. (DOCX) [file pone.0212524.s008.docx]

# S7 Table. Hemi- desmosomes per length based on Transmission electron microscopy micrographs

Sample names with uneven numbers (grey background) represent large (3 mm) explants. Even numbers mean small (1 mm) explants.

| Sample | Length(µm) | Number of hemi-desmosomes | Hemi-desmosomes/µm |
| --- | --- | --- | --- |
| b1 | 47.4 | 81 | 1.71 |
| b2 | 27.43 | 60 | 2.19 |
| b3 | 28.18 | 32 | 1.14 |
| b5 | 60.49 | 49 | 0.81 |
| b6 | 56.55 | 81 | 1.43 |
| B1 | 22.61 | 50 | 2.21 |
| B2 | 28.02 | 33 | 1.18 |
| B3 | 27.74 | 30 | 1.08 |
| B5 | 28.87 | 37 | 1.28 |
| B6 | 28.69 | 39 | 1.36 |
| B7 | 28.16 | 25 | 0.89 |
